# Interaction between gut microbiota and immune checkpoint inhibitor-related colitis

**DOI:** 10.3389/fimmu.2022.1001623

**Published:** 2022-10-27

**Authors:** Guanzhou Zhou, Nana Zhang, Ke Meng, Fei Pan

**Affiliations:** ^1^ Department of Gastroenterology and Hepatology, The First Medical Center, Chinese PLA General Hospital, Beijing, China; ^2^ School of Medicine, Nankai University, Tianjin, China; ^3^ Medical School of Chinese PLA, Beijing, China

**Keywords:** immune checkpoint inhibitor, gut microbiota, colitis, diarrhea, microbiome

## Abstract

Immune checkpoint inhibitors (ICIs) have become a promising therapeutic strategy for malignant tumors, improving patient prognosis, along with a spectrum of immune-related adverse events (irAEs), including gastrointestinal toxicity, ICI-related colitis (IRC), and diarrhea. The gut microbiota has been suggested as an important regulator in the pathogenesis of IRC, and microbiota modulations like probiotics and fecal microbiota transplantation have been explored to treat the disease. This review discusses the interaction between the gut microbiota and IRC, focusing on the potential pathogenic mechanisms and promising interventions.

## Introduction

Immune checkpoint inhibitors (ICIs) have received great attention as they have rapidly altered the treatment landscape for multiple tumors, including lung cancer, metastatic melanoma, and urinary epithelial carcinoma. ICIs block inhibitory molecules, such as cytotoxic T-lymphocyte-associated protein 4 (CTLA-4), programmed cell death protein 1 (PD-1) and its ligand 1 (PD-L1) and enhance anti-tumor T-cell activity providing clinical benefits in many patients with advanced cancers (1–3). Yet, multiple organs like skin, lung, liver, and digestive tract are susceptible to the unrestrained immune response activation by the utility of ICIs, which developed to the immune-related adverse events (irAEs) ultimately, including ICI-related colitis (IRC) and diarrhea, which are major causes of ICI discontinuation (4–6).

Studies have suggested that the occurrence of diarrhea and colitis is associated with the ICI used. For example, Tandon et al. performed a meta-analysis to evaluate the risk of colitis and diarrhea in patients with advanced melanoma treated with ICIs (anti-PD-1 or anti-CTLA-4 therapy) and concluded that diarrhea and colitis are more frequent in patients treated with CTLA-4 inhibitors (7). Another study showed that patients treated with anti-CTLA-4 therapy have a higher rate of diarrhea (31.8% in anti-CTLA-4 alone versus 10.5% in anti-PD-1 alone) and colitis (7.7% in anti-CTLA-4 alone versus 0.8% in anti-PD-1 alone); also, diarrhea seems to be more common in patients treated with dual ICI therapy than in those with a single-ICI agent (8). One possible explanation for this preference is that the CTLA-4 receptor is often expressed on the surface of CD4^+^ and CD8^+^ cells, subsets of B cells and thymocytes, resulting in inhibition at the initial step in an immune response while the PD-1 and its ligand blockades aim at late T-cell proliferation, causing a more localized immune reaction (9, 10).

Yet, the mechanisms of IRC are still not fully understood and several key aspects have been proposed: (a) the cross-reactivity of the common antigens on tumor and healthy tissues; (b) activation of humoral immunity like elevated pre-existing autoantibodies level; (c) modulation of pro (anti)-inflammatory cytokines; (d) enhanced complement-mediated inflammation; (e) regulation of effector or suppressor immune cells (10, 11). Moreover, different management is proposed based on the IRC severity. Mild or moderate IRC is closely observed and applied with supportive treatment. Higher-grade toxicities cases may discontinue the ICI course and receive corticosteroids or immunosuppressive therapies such as tumor necrosis factor-α (TNF-α) inhibitors (e.g., infliximab) and anti-integrin agents (e.g., vedolizumab) (11). Recent studies have highlighted an indispensable role of the gut microbiota in the communication between ICI and patients. The anticancer immunotherapy relies on the immunization with some species like *Bacteroides fragilis* (12). *Bifidobacterium* and *Faecalibacterium* promote ICI efficacy with augmented dendritic cell function and T cell accumulation in the tumor microenvironment (13, 14). Fecal microbiota transplantation (FMT) has also demonstrated the ability of overcoming resistance to anti-PD-1 therapy in melanoma patients (15). Besides, emerging evidence emphasizes the critical involvement of gut microbiota in the pathogenesis of IRC, patients vulnerable to IRC development seem to have a distinct microbiota profile (**Table 1**) (16–22) and the microbiota modulation offers a novel alteration for the treatment. This review discusses the interaction between the gut microbiota and ICI-related colitis, focusing on the potential pathogenic mechanisms and promising interventions.

**Table 1:** Gut microbiota studies for immune checkpoint inhibitor-related colitis and other irAEs.

Study	Country	Sample size	Study period	Drugs	Sample type	Incidence	Main findings
Chaput et al. (16)	France	MM (n=26)	2013.3-2014.12	Anti-CTLA-4 (n=26)	Fecal	Colitis (n=7)	Most of the baseline colitis-associated phylotypes were related to Firmicutes, whereas no colitis-related phylotypes were assigned to Bacteroidetes.
Dubin et al. (17)	the USA	MM (n=34)	Not available	Anti-CTLA-4 (n=34)	Fecal	Colitis (n=10)	Bacteroidetes phylum and three of its families (Bacteroidaceae, Rikenellaceae, Barnesiellaceae) had higher abundance in colitis-free patient.
Usyk et al. (18)	the USA	Advanced stage melanoma (n=27)	2016.9-2017.11	Anti-PD-1 (n=12);	Fecal	IrAEs:	Patients with high abundance of *Bacteroides dorei* at baseline have high risk for severe irAEs, while patients characterized by high abundance of *Bacteroides vulgatus* have low risk.
				Combined (n=15)		Not applicable	
Mohiuddin et al. (19)	the USA	Stage III and IV melanoma (n=568)	2018-2019	Anti-CTLA-4 (n=232);	Fecal	Antibiotic group: colitis (n=11); None-antibiotic group: colitis (n=20);	The antibiotic group had a greater incidence of colitis
				Anti-PD-1 (n=286);			
				Combined (n=50)			
Zhao et al. (20)	China	Lung cancer (n=100);	2018.8-2020.7	Nivolumab (n=52);	Fecal	IrAEs:	Antibiotic exposure was associated with a higher risk of irAEs
		Esophagus cancer (n=32);		Pembrolizumab (n=56);		Lung cancer (n=25); Esophagus cancer (n=8);	
		Gastrointestinal cancer (n=24);		Camrelizumab (n=40);		Gastrointestinal cancer (n=6);	
		Others (n=12)		Toripalimab (n=20)		Others (n=3)	
Liu et al. (21)	China	NSCLC (n=102);	2018.10-2021.3	Anti-PD-1 (n=150)	Fecal	Severe diarrhea (n=3);Mild diarrhea (n=10)	Patients with severe diarrhea showed a higher level of *Stenotrophomonas* and *Streptococcus* compared with patients without irAEs or with mild diarrhea
		Nasopharyngeal carcinoma (n=7);					
		Melanoma (n=5);					
		Esophagus cancer (n=5);					
		Others (n=31)					
Mao et al. (22)	China	Unresectable HCC (n=30); Advanced BTC (n=35)	2018.11-2020.12	Anti-PD-1 (n=65)	Fecal	Severe diarrhea (n=8);	Patients with severe diarrhea tended to have decreased gut microbiome diversity and relative abundance; *Prevotellamassilia timonensis* was observed in more severe diarrhea patients
						Mild diarrhea or absence (n=57)	

MM, metastatic melanoma; irAEs, immune related adverse events; NSCLC, non-small-cell lung carcinoma. HCC, hepatocellular carcinoma; BTC, biliary tract cancer.

## The composition of gut microbiota on ICI-related colitis

Accumulating studies indicate that the gut microbiota signature has a strong link with IRC. Chaput et al. (16) collected fecal samples from twenty-six metastatic melanoma patients before the ICI therapy and analyzed the gut microbiota 16S rRNA gene sequencing data. According to the characteristics of baseline microbiota composition, patients were divided into 3 clusters. There was a high proportion of *Faecalibacterium* and other *Firmicutes* in the microbiota composition of patients belonging to Cluster A. Cluster B was enriched in *Bacteroides*, and Cluster C, *Prevotella*. At the phyla level, patients in Cluster A were prone to develop colitis, with a preference of Firmicutes, while patients without colitis had more Bacteroidetes (like Cluster B). Specifically, *Bacteroides vulgatus*, and *Faecalibacterium prausnitzii A2-165* were detected as potential biomarkers for colitis absence during ICI therapy, whereas several OTUs in Firmicutes phylum, and *Gemmiger formicilis ATCC 27749* were detected to be with increased risk of colitis. Meanwhile, there is an overlap that gut microbiota composition associated with IRC also promotes ICI clinical response. For example, *Faecalibacterium* magnifies systemic immune response mediated by up-regulated antigen presentation and intensified effector T cell function. These overactive immune cells not only infiltrate in tumor microenvironment, strengthening ICI anti-tumor effect, but attack normal intestinal mucosal and induce IRC. In another study of advanced-stage melanoma patients undergoing ICI, stool samples were collected before, during, and after the treatment. Two natural gut microbiome clusters with distinct profiles were identified, and patients with a high proportion of *Bacteroides dorei* in gut microbiota had high risk of irAE, while the *Bacteroides vulgatus* was identified as a specific dominance strain in the low-risk cluster (18). Apart from the specific strain, it is inferred that the IRC is associated with decreased diversity of gut microbiome. The low richness of abundance in gut microbiota often refers to a fragile immune homeostasis, which are easily perturbed by ICIs intervention as observed in IRC patients. Mao et al. (22) displayed that ICI-treated hepatobiliary cancer patients with severe diarrhea tends to have lower phylogenetic diversity of gut microbiota. They also recognized several enriched taxa with significant differentiation between the severe and mild diarrhea groups. The enrichment of *Dialister* genus, which belongs to the Firmicutes phylum, was observed in the mild group. Notably, severe diarrhea patients had a higher abundance of *Prevotellamassilia timonensis*, which has been suggested as valuable biomarker. Overall, it could be speculated that a higher diversity of gut microbiome may be a protective factor against IRC.

## Antibiotic use on ICI-related colitis

Patients with malignant tumor tend to experience infection due to their impaired immune system, causing higher exposure to antibiotics. In clinical practice, about 70% cancer patients receive antibiotics during the ICI treatment, how they affect IRC deserves exploration (23). Epidemiological studies emphasized that antibiotic therapy weakens ICI efficacy and shortens patient survival across malignancies (24). Antibiotics alter the composition of gut microbiota, leading a decreased bacterial-mediated secondary bile acids production and an increased inflammasome signaling, thus promotes a pro-inflammatory state, susceptible to IRC (25). As a result, the history of antibiotic use may be an indicator of IRC. Researchers established an ICI-related colitis mice model by combining dextran sulfate sodium (DSS) and anti-CTLA-4 to simulate the inflammation condition. Compared to the control group (with ICI isotype and DSS), mice with anti-CTLA-4 pretreatment showed higher mortality, more body weight loss, and worse histopathological scores, thus declaring that preprocess of ICI exaggerates the DSS-induced inflammation in mice. Moreover, pretreatment with vancomycin provoked an even more severe, largely fatal form, indicating that a Gram-positive component of the microbiota had a mitigating effect on colitis (26). Due to the limitation of mice models, they generally do not develop colitis after ICI treatment, unlike malignancy patients, in the absence of chemical damage or genetic defects. Therefore, the potential influence of additional DSS process requires to be further explored.

A clinical observational study including 832 patients with ICI treatment exhibited that antibiotic exposure is strongly correlated to grade 3 or 4 irAEs (20). Mohiuddin et al. (19) investigated 568 patients with stage III and IV melanoma receiving immunotherapy. Patients treated with antibiotics within 3 months prior to the first infusion of ICI had significantly worse overall survival and a greater incidence of colitis. The incidence and severity of colitis varies according to some factors. Anaerobic antibiotics were associated with expanded immunosuppressant use, hospitalization, intensive care unit admission due to IRC, and elevated severity grades. At the onset of colitis, the empirical antibiotic group had a higher recurrence rate and colitis severity than the group receiving antibiotics when there was positive evidence of infection. Antibiotic therapy changed the microbiome taxonomic diversity profoundly, inducing a loss of protective bacteria and an impaired immune homeostasis, thus with a worse prognosis. Therefore, it provides an implication for clinical practice that antibiotic use should be taken into consideration carefully in cancer patients.

## Potential mechanisms of interaction between gut microbiota and ICI-related colitis

**Figure 1:**
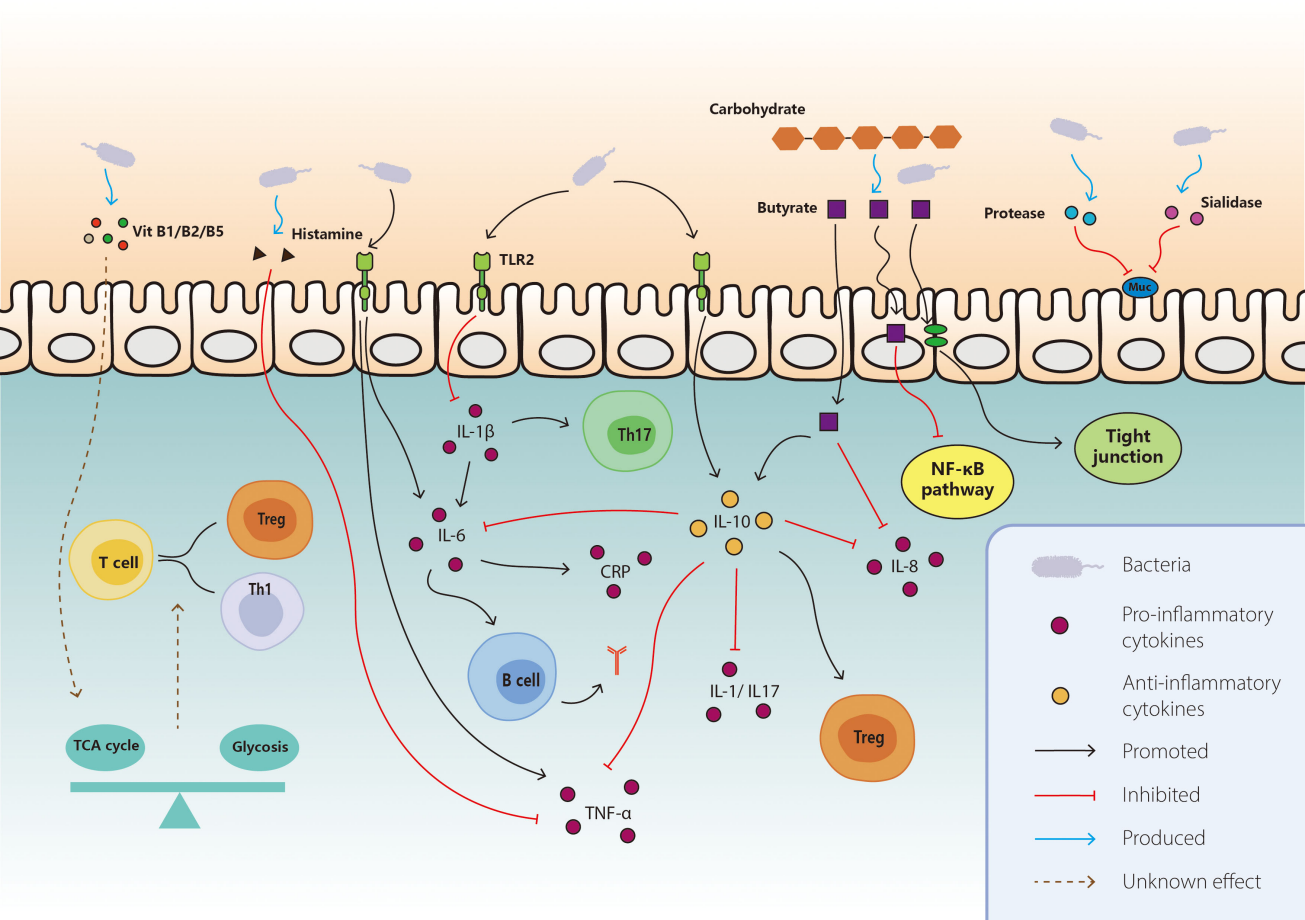
Mechanisms of interaction between gut microbiota and ICI-related colitis. On the basis of the known interaction between gut microbiota and IRC, the main mechanisms included direct effect, metabolites, cytokines, and immune cells. For protective bacteria, the pro-inflammatory pathways like IL-1b and TNF-a are inhibited, together with promoted anti-inflammatory pathways including IL-10, Th17 cells, and Treg cells. They also modulate the differentiation of T cells through vitamin B and tricarboxylic acid cycle. Butyrate produced by bacteria exerts anti-inflammatory effect via various aspect like consolidating tight junction, inducing IL-10 and suppressing NF-κB. As for harmful bacteria, they secrete some enzymes to destruct mucin and enhance the pro-inflammatory pathway like IL-6, TNF-a, CRP, and antigen production. TCA, tricarboxylic acid; NF-kB, nuclear factor kappa B; TLR, toll-like receptor; TNF-a, tumor necrosis factor-a; IL, interleukin; CRP, C-reactive protein.

The species and diversity of gut microbiota influence the development of IRC; yet, the underlying mechanism is still unclear. Deciphering the biological mechanisms is critical for optimizing patient outcome. Multiple results highlighted the involvement of gut microbiota in IRC pathogenesis, not only through direct effect of bacteria, but also through indirect mechanisms like regulating metabolites, cytokines and immune cells. It provides a better understanding of the disease and some novel targets for intervention. This part depicts early evidences and hypothetical scenarios, then discusses the potential mechanisms of the interaction between gut microbiota and ICI-related colitis (**Figure 1**).

### Direct effect of bacteria

Mounting evidences illustrated that the bacteria exert direct effect *via* extracellular enzymes, lipopolysaccharide (LPS) and others in their interaction with IRC. Higher levels of *Stenotrophomonas* have been found in severe diarrhea patients receiving ICI treatment (21). *Stenotrophomonas* is considered an environmental bacterium commonly found in the respiratory or digestive tract. It often causes pulmonary diseases like Stenotrophomonas maltophilia pneumonia and diarrhea or enteritis in some cases (27, 28). Malignancy patients with impaired immunity are predisposed to this strain and tend to experience severe diarrhea or IRC if infected (27). A range of extracellular enzymes by *Stenotrophomonas maltophilia*, including DNase, RNase, lipases, protease, and elastase, may be key factors in pathogenesis. Assisted with these enzymes, the strain breaks down the tight junction, decomposes mucin, invades tissue and causes IRC. Bacterial enzymes also play a critical role in the pathogenesis of *Prevotellmassilia timonensis*, a subspecies strain of *Prevotella*, which is associated with severe diarrhea in ICI-treated patients. It secretes sialidase, breaks sialic acid and degrades the mucin, increasing the intestinal barrier permeability (29). Dendritic cells (DCs) are also involved in its pathogenic mechanism (30). Endotoxin-like lipopolysaccharide (LPS) is another virulence factor promoting the inflammation. It actives immune cells through the toll-like-4 receptors, synthesizes and releases a variety of cytokines and inflammatory mediators, causing inflammation (31, 32). Compared to the control group, the LPS level was reduced in serum and feces of mice fed with *B. vulgatus*, which has a strong correlation with few irAEs, indicating a potential protective mechanism *via* LPS reduction (33). Microbial anti-inflammatory molecules (MAMs) have same favorable effects, which contain a series of proteins produced by *Faecalibacterium prausnitzii*. In animal models, MAMs exhibit anti-inflammatory effect by blocking the NF-κB pathway and inhibiting the pro-inflammatory Th1 and Th17 immune responses. It also consolidates the gut barrier by upregulating the tight connection gene Zo-1 (34, 35). Therefore, *Faecalibacterium prausnitzii* could prevent patients from IRC and act as a biomarker for colitis absence.

### Metabolites

#### Short-chain fatty acid (SCFA)

The gut microbiota consumes carbohydrates and produces variable bioactive molecules, modulating the host immune system differently (36). SCFAs are one of the most extensively characterized classes of microbial metabolites (37, 38). Bacteria break complicated carbohydrates into simple fatty acids like acetate, propionate, and butyrate. These small molecules supply energy for intestinal epithelial cells and exert diverse effects on immune cell function and cytokine production (39). The anti-inflammation characteristic of butyrate is partly attributed to inhibiting the NF-κB activation and its downstream pathway, which in turn reduces the pro-inflammatory cytokines, such as IL-8, and increases anti-inflammatory factors like IL-10. The butyrate also induces tight connection protein expressions in the mucosa and consolidates the gut barrier (40). Indeed, a higher abundance of butyrate-producing *Faecalibacterium prausnitzii A2-165* was detected in colitis-absent patients with ICI therapy compared to those who experienced colitis (16). On the contrary, the reduction of SCFAs cannot supply the cell with enough energy, resulting in an impaired gut barrier and immune system. Some species of *Prevotella* genus aggravate local and systemic inflammation *via* reduction of SCFAs and IL-18 (41), which may explain their enrichment in feces of severe diarrhea patients receiving ICI treatment for malignancy.

#### Vitamin and polyamine

Dubin et al. (17) demonstrated that bacteria belonging to the Bacteroidaceae, Rikenellaceae, Barnesiellaceae family are enriched in patients resistant to IRC. Furthermore, according to the shotgun sequencing and metabolic pathway reconstruction, genetic pathways involved in vitamin B biosynthesis and polyamine transport are correlated with an absence of colitis.

Vitamins are necessary micronutrients generated by plants and bacteria. The gut microbiota can metabolize vitamins for humans through its relevant enzymes and transporters (42). Vitamin B1 (thiamine) is essential in energy metabolism, especially in the tricarboxylic acid (TCA) cycles (43). Accumulating evidence proved an energy supply balance between glycolysis and the TCA cycle for immune cells. Generally, quiescent or regulatory-type cells (e.g., naive T cells, Treg cells, and M2 macrophages) use the TCA cycle for energy generation, whereas activated or pro-inflammatory cells (e.g., Th1, Th2, Th17, and M1 macrophages) rely on glycolysis (44, 45). Therefore, thiamine regulates the immune cell balance and poses a potential effect on the IRC. Vitamin B2 (riboflavin) and its active forms (flavin adenine nucleotide (FAD) and flavin mononucleotide (FMN)) are cofactors in enzymatic reactions in the Krebs cycle and fatty acid oxidation (43). The oxidation process is involved in the activation, differentiation, and proliferation of immune cells *via* producing acetyl-CoA for TCA cycles and energy generation, while riboflavin deficiency inhibits acyl-CoA dehydrogenase activity in the process (46). It is speculated that riboflavin modulates immune function through fatty acid oxidation. Moreover, in the presence of NADPH oxidase 2, riboflavin induces reactive oxygen species (ROS) production, which is an essential effector and signaling molecule in inflammation and immunity (47). Pantothenate, also known as vitamin B5, is a precursor of coenzyme A (CoA). Similar to thiamine and riboflavin, pantothenate has a crucial effect on immunity *via* cell energy consumption as coenzyme A is an indispensable cofactor for the TCA cycle and fatty acid oxidation (43).

Polyamines are small cationic amines exported from bacterial cells *via* the spermidine and putrescine transport systems (pot A, B, C, and D). It resists inflammation partly by promoting colonic epithelial cell proliferation to maintain the epithelial barrier (48). Spermine, produced by amino acid decarboxylation, reduces colonic IL-18 levels and inhibits NLRP6 inflammasome assembly (49). It also suppressed the secretion of pro-inflammatory cytokines like TNF-α and lymphocyte function-associated antigen-1 (LFA-1), which is a regulator of immune cell adhesion and migration (50).

#### Conjugated linoleic acid

Conjugated linoleic acid (CLA) is a group of 18 carbon conjugated dienoic acids. It is reported to benefit local immunomodulatory activity through up-regulating anti-inflammation factors, inhibiting pro-inflammation factors, and improving the tight junctions. Some studies displayed that human commensal bacteria like *Bifidobacterium* possess CLA-production ability and exhibit anti-inflammation ability (51). Wall et al. (52) found that some isomers of CLA are elevated in murine fed with *Bifidobacterium breve NCIMB 702258*, meanwhile, some pro-inflammatory cytokines like TNF-α and IFN-γ were decreased. Another subtype of *Bifidobacterium breve* ameliorated mice colitis through CLA accumulation, along with advanced tight conjunction, elevated mucin and decreased IL-1 and IL-6 (53).

### Cytokines

Cytokines are a series of small molecules mainly produced by immune cells. They modulate cell growth, differentiation, development and apoptosis, regulate immunity and contribute greatly to multiple bio-active responses including inflammation. Microorganisms induce human cell to generate considerable cytokines, which mainly consists of two types, the pro-inflammatory and anti-inflammatory cytokines. For unfavorable bacteria, they promote the level of inflammation-promotion cytokines like IL-6, TNF-α and IL-1β, exaggerating the IRC. Meanwhile, some favorable bacteria support the anti-inflammatory production like IL-10, beneficial for IRC.

#### IL-6

IL-6 is one of the most essential and well-studied pro-inflammatory cytokines, enabling B cells to proliferate, differentiate, and secrete antibodies, and inducing a series of acute-phase reaction proteins such as C reactive protein, serum amyloid A, thrombopoietin, and complement C3. In mice models, pretreatment of ICI process enhanced the susceptibility of DSS-Induced colitis, accompanied by exacerbated hyperplasia and ulceration. It also raised inflammatory leukocyte infiltration in colonic sections, as well as the levels of inflammatory cytokines, IL-6, TNF-α, and IFN-γ in the circulation (54). Mounting evidences highlight the strong association among IL-6, bacteria and colitis. The relative abundance of *Streptococcus* in feces has a positive correlation with serum IL-6 level in mice models of colitis and with colonic mucosal TLR2 receptor expression in ulcerative colitis patients, respectively (55, 56). Moreover, another study manifested elevated levels of IL-6 and TNF-α in the serum of mice infected with *Streptococcus via* a TLR2 receptor-dependent pathway (57). Compared to control mice, *Bacteroides*-treated mice exhibited suppressed inflammation response and significantly lower plasma levels of pro-inflammatory cytokines, such as IL-6, IFN-γ, and TNF-α (33). Overall, it is believed that IL-6 meditates pathogenicity of bacteria on colitis and the reduction of IL-6 might contribute to the resistance to the IRC.

#### TNF-α

Apart from IL-6, another possible pathogenic mechanism of *Streptococcus* on IRC is TNF-α induction, meditated by primary bile acid and its receptors (58). TNF-α regulates multiple cellular responses such as vasodilation, edema formation, and leukocyte-epithelial cell adhesion. It also meditates blood coagulation and promotes oxidative stress, causing fever and inflammation indirectly (59). Conversely, the reduction of TNF-α contributes to recovery from colitis. In children with active distal ulcerative colitis, rectal infusion of *Lactobacillus reuteri* reduces TNF-α mucosal expression (60). The bacteria decompose dietary L-histidine to generate histamine, stimulate intracellular cAMP production through H2 receptors, inhibit TNF-α production in a PKA-MEK/ERK-MAPK-dependent pathway and relieve mucosal inflammation effectively (61).

#### IL-1β

As a key pro-inflammatory cytokine, IL-1β is engaged in various autoimmune inflammatory responses and cellular activities, including cell proliferation, differentiation, and apoptosis. It is confirmed that *Prevotella* aggravates the colitis *via* meditating the maturity of IL-1β (62). *Bacteroides intestinalis* was also proved to induce IRC *via* up-regulating IL-1β mucosal transcription (63). This cytokine activates the release of other pro-inflammatory cytokines like IL-6 and induces the differentiation of the Th17 cells. It also promotes monocytes differentiation to conventional DCs and M1-like macrophages and supports the activated B lymphocytes to proliferate and differentiate into plasma cells (64, 65). Meanwhile, the inhibition of IL-1β might contribute to the anti-inflammatory effect of *Bifidobacterium breve* through the interaction with TLR2 receptor and NF-κB pathway blocking (66).

#### IL-10

As for anti-inflammation cytokines, IL-10 suppresses the expression of major histocompatibility complex II (MHC II) on the surface of monocytes, restrains its antigen presentation, impairs the activity of T lymphocytes, and prohibits the activation, migration, and adhesion of inflammatory cells. Moreover, it strongly depresses the synthesis of IL-1, IL-6, IL-8, TNF-α, granulocyte-macrophage colony-stimulating factor (GM-CSF), and granulocyte colony-stimulating factor (G-CSF) at the transcriptional level, leading an anti-inflammatory effect (67, 68). IL-10 also antagonizes the IL-17 and increases the proportion of Foxp3^+^ Treg cells in CD4^+^ T cells (69). The special cytokine contributes greatly to bacteria protection against colitis. After supplementation with *Bifidobacterium breve* for mice, the expression of IL-10 and IL-10Ra expanded in Treg cells in the lamina propria of the intestinal mucosa, which prevents effector T cell proliferation. However, the colitis-relieving effects of *B. breve* were reduced after IL-10 receptor knockout in mice, emphasizing the role of IL-10 in the anti-inflammatory effects of *B. breve* (70). The strain activates intestinal CD103^+^ DCs through the TLR2/MyD88 pathway to generate IL-10 and induce IL-10-secreting type 1 regulatory T cells in the colon, which in turn induces IL-10 and TGF-β, weakening Th1 and Th2 cells function and ameliorating the colitis (71). Other studies pointed out that *F.prausnitzii A2-165* attenuates mice colitis induced by 2,4,6-trinitrobenzene sulfonic acid (TNBS) or dinitrobenzene sulfonic acid (DNBS) and modulates the T cell response *via* inducing IL-10 in human and murine dendritic cells (72–74). Increased IL-10 levels were also observed in mice fed with *Lactobacillus reuteri*, accompanied by inflammation remission and IL-17 and IL-23 reduction (54). In the future, the level of serum IL-10 may predict patients’ risk for IRC and reflect the efficacy of treatment.

### Immune cells

Normally, immune checkpoint inhibitors raise the T cell activity against antigen presented in tumor. Sometimes, the activated immune cells target healthy tissues which have the same antigen causing inflammation like IRC. In general, the enrichment of pathogenic bacteria in IRC patient is usually accompanied with effector T cell accumulation. For those favorable strains for IRC, the immunosuppressive properties of Treg cell enable them to exert fundamental impact on anti-inflammation, partly contributing to their protection. Treg cells are necessary component of immune cells, responsible for maintaining self-tolerance and avoiding excessive immune response damage to the body. Treg cells moderate immunity partly by blocking the induction of IL-2 production in responder T cells and that both IL-10 and TGF-β are engaged in the process (75). Another mechanism of regulation is cytolysis of target cells mediated by Treg cells, which relies on granzyme A and B in human (76). Wang et al. (26) found that the supplementation of *bifidobacterium* mixture reduces the IRC inflammation and this effect seems to be dependent on Treg cells. Further research identified the effective specific strain, *Bifidobacterium breve*, and proved that the immune modulation of the strain on IRC has a close association with Treg cell energy metabolism (70). After gavage with *B. breve*, the circulation level of suberic acid in mice was significantly increased, reflecting the enhanced mitochondrial activity, along with elevated mitochondrial volume and stress level of Treg cells in the lamina propria. Consistent with this finding, multiple genes related to mitochondrial structural components and function were obviously upregulated (70). The relative increase in the proportion of Treg cells within the colonic mucosa was also presented in a refractory IRC patient who achieved recovery after receiving FMT therapy (77). Therefore, the relative abundance of Treg cells could be a predictor for colitis absence and a therapy target in the future.

## A promising therapy for ICI-related colitis

The gut microbiota occupies a substantial place in the pathogenesis of IRC, which presents an applicable therapy through modulating its composition. Recently, probiotic supplementation has been recommended for IRC. *B.breve* exhibited anti-inflammatory effect in mice models, it ameliorates their immunopathological condition and rescues them from weight loss without apparent influence on anti-tumor immunity. *Lactobacillus reuteri* and *Lactobacillus rhamnosus GG* both abrogated IRC by inhibiting group 3 innate lymphoid cells (ILC3s) or regulating T cells (54, 78). FMT was introduced into the management as it manipulated the gut microbiota of recipients from donor microorganisms and small molecules like SCFAs. Recently, the therapy has been utilized on two refractory IRC patients (77). Two patients both received systemic corticosteroids, infliximab, and vedolizumab but had no settlement of symptoms. After the transfusion from an unrelated donor, they achieved marked improvements both in clinical symptoms and on endoscopic evaluation, with reduced inflammation and resolved ulcerations. Further analyses of patient's microbial composition revealed a tendency towards that of donor. The proportion of immune cells infiltrated in the colonic mucosa changed after the transplantation, such as the reduction in CD8^+^T cells, providing a plausible explanation of FMT treatment on ICI-related colitis. Additional cases encouraged the idea that FMT appears to be a promising option for ICI-related colitis patients resistant to corticosteroids and monoclonal antibody therapies (79, 80). Besides, a clinical trial is undergoing about FMT in treating ICI induced-diarrhea or colitis in genitourinary cancer patients (NCT04038619). However, further investigations are required to verify the efficacy and safety of FMT on ICI-related colitis, like the donor selection and transplant frequency.

## Conclusion

Alterations and dysbiosis of gut microbiota have strong association with immune-related adverse events caused by ICIs, particularly the ICI-related colitis. Several strains have been proposed as valuable biomarkers of IRC. Studies have also suggested that microbiome dysbiosis caused by antibiotics may be an indicator of IRC. Moreover, multiple factors have been identified as involved in this pathogenesis, including metabolites, cytokines, and immune cells. Until now, there is no consensus about the exact role of one strain on IRC and different results are presented based on small sample studies. Therefore, studies with large sample and detailed mechanism are required. Regarding potential treatments, microbiota modulations such as probiotics and fecal microbiota transplantation have been explored as a promising therapy for ICI-related colitis.

## Author contributions

FP contributed to the conception and design of the review. The first draft of the manuscript was written by GZ. GZ created all the Figures and tables. NZ, KM, and FP revised the manuscript. All authors contributed to the article and approved the submitted version.

## Conflict of interest

The authors declare that the research was conducted in the absence of any commercial or financial relationships that could be construed as a potential conflict of interest.

## Publisher’s note

All claims expressed in this article are solely those of the authors and do not necessarily represent those of their affiliated organizations, or those of the publisher, the editors and the reviewers. Any product that may be evaluated in this article, or claim that may be made by its manufacturer, is not guaranteed or endorsed by the publisher.
